# A Novel Bilayer Polycaprolactone Membrane for Guided Bone Regeneration: Combining Electrospinning and Emulsion Templating

**DOI:** 10.3390/ma12162643

**Published:** 2019-08-20

**Authors:** Betül Aldemir Dikici, Serkan Dikici, Gwendolen C. Reilly, Sheila MacNeil, Frederik Claeyssens

**Affiliations:** 1 Department of Materials Science and Engineering, University of Sheffield, Kroto Research Institute, Sheffield S3 7HQ, UK; 2 Department of Materials Science and Engineering, University of Sheffield, INSIGNEO Institute for in silico Medicine, The Pam Liversidge Building, Sheffield S1 3JD, UK

**Keywords:** guided tissue regeneration (GTR), guided bone regeneration (GBR), barrier membrane, polyHIPE, electrospinning, polymer, polycaprolactone, CAM assay, tissue engineering, dental biomaterials

## Abstract

Guided bone regeneration is a common dental implant treatment where a barrier membrane (BM) is used between epithelial tissue and bone or bone graft to prevent the invasion of the fast-proliferating epithelial cells into the defect site to be able to preserve a space for infiltration of slower-growing bone cells into the periodontal defect site. In this study, a bilayer polycaprolactone (PCL) BM was developed by combining electrospinning and emulsion templating techniques. First, a 250 µm thick polymerised high internal phase emulsion (polyHIPE) made of photocurable PCL was manufactured and treated with air plasma, which was shown to enhance the cellular infiltration. Then, four solvent compositions were investigated to find the best composition for electrospinning a nanofibrous PCL barrier layer on PCL polyHIPE. The biocompatibility and the barrier properties of the electrospun layer were demonstrated over four weeks in vitro by histological staining. Following in vitro assessment of cell viability and cell migration, cell infiltration and the potential of PCL polyHIPE for supporting blood vessel ingrowth were further investigated using an ex-ovo chick chorioallantoic membrane assay. Our results demonstrated that the nanofibrous PCL electrospun layer was capable of limiting cell infiltration for at least four weeks, while PCL polyHIPE supported cell infiltration, calcium and mineral deposition of bone cells, and blood vessel ingrowth through pores.

## 1. Introduction

Periodontal regenerative procedures require the use of guided tissue regeneration/guided bone regeneration membranes (GTR/GBR) in various conditions such as socket preservation, grafting, maxillary sinus elevation and the treatment of chronic periodontitis [1]. The main principle of the GTR/GBR procedure is to place a barrier membrane (BM) between epithelial tissue and bone or bone graft to prevent migration of the fast-proliferating epithelial cells into the defect site to be able to preserve a space for infiltration of bone cells into periodontal defect site [2,3].

The earliest developed membranes were made of non-resorbable materials such as cellulose filters, polytetrafluoroethylene, and titanium meshes, but the necessity of second surgery for removal led to the development of resorbable membranes [4]. The most common natural membranes are made of porcine, bovine, or human collagen. Despite their high biocompatibility, the main disadvantages of collagen membranes are their potential for antigenicity, poor mechanical properties, and rapid degradation [5,6]. Alternatively, synthetic polymers such as polyglycolic acid and polylactic acid have been commonly investigated for the fabrication of BMs. Although they are biodegradable and non-cytotoxic, their rapid degradation can generate an acid environment around the implant, which may cause adverse inflammatory tissue reactions [7,8].

Polycaprolactone (PCL) is another biocompatible and bioresorbable synthetic polymer, which degrades more slowly and consequently does not produce an overly acidic environment in the degradation process [7]. United States Food and Drug Administration (FDA) approved biomedical devices made of PCL are already on the market, which makes PCL a promising material for other biomedical applications. Additionally, due to its ease of fabrication in different forms, PCL is used as a scaffold material for both hard and soft tissue engineering [9]. It has previously been reported for various biomedical applications including drug delivery applications [10,11], periodontal regeneration [12,13], vascular grafts [14], bone tissue engineering applications [15,16], and wound healing applications [17,18]. One of the main drawbacks of PCL, as with many other synthetic polymers is that it is hydrophobic, which limits the polymer–cell interaction [19,20]. Plasma treatment is one of the most common and effective ways to promote hydrophilicity of the polymer surfaces by adding polar groups to the surface of the material without altering the bulk properties [21,22,23,24,25].

A BM is expected to be in contact with both hard and soft tissues, and it has different functions on each side. While being cell occlusive on the side in contact with soft tissue, it should encourage bone regeneration on the other side. There are many methods used in barrier membrane fabrication; such as solvent casting [26,27], electrospinning [13,28,29,30,31], phase inversion, freeze-drying [32,33], and 3D printing [34,35].

Electrospinning is a simple, rapid and versatile technique for fabricating fibres with varying diameters from a few nanometres to several micrometres from a wide variety of materials [36]. PCL is also one of the widely-used polymers, which has been electrospun for its use in numerous applications [37]. Several solvents and solvent blends have been reported to be used to dissolve PCL for preparing the electrospinning solution [38]. Although several parameters have been associated with the size of fibres [39], the composition and the ratio of solvents have been demonstrated to have a significant effect on fibre diameters [40]. As electrospun nanofibres are shown to prevent cell infiltration without limiting the diffusion of oxygen and nutrients [22], electrospinning is a promising method to manufacture a physical barrier.

Emulsion templating is another scaffold manufacturing technique where polymer solution and water are mixed in the presence of surfactants to form an emulsion. When the water droplets are encapsulated in a polymer solution, it is called water in oil (w/o) emulsion. If the internal phase volume (water content) is increased over 74% (v/v), the emulsion is classified as high internal phase emulsion (HIPE) [41,42,43,44]. After solidification of the polymer phase (continuous phase) by thermal curing or photo-curing or solvent evaporation, the structure is locked, and water droplets are removed. The resulting porous structure is defined as polymerised HIPE (polyHIPE). PolyHIPEs are favourable as tissue engineering scaffolds because of their highly interconnected porous structures which have been previously demonstrated as promoting cell migration and tissue ingrowth [45,46,47,48,49,50].

Manufacturing of scaffolds made of photocurable PCL by using emulsion templating technique is challenging because of the high viscosity of the polymer, which constrains the mixing of two phases during emulsion formation [51,52,53,54]. We have recently developed and reported a production route of polyHIPEs made of photocurable PCL and showed the biocompatibility of the material by using human dermal fibroblasts [46]. However, this developed composition has not been used for any specific application yet, and the use of emulsion templated polyHIPEs in GBR/GTR barrier membrane applications has not previously been reported.

In this study, we combined two methods, emulsion templating and electrospinning, to manufacture a bilayer, bioresorbable BM made of polycaprolactone. Emulsion templating is selected for manufacturing of the layer, which will be in contact with bone/bone graft. 250 μm thick PCL polyHIPE layer was manufactured and treated with air plasma to enhance the cellular infiltration. Following the in vitro evaluation of the biological performance, the suitability of PCL polyHIPE morphology for blood vessel infiltration through the pores was further investigated using an ex-ovo chick chorioallantoic membrane (CAM) assay. Electrospinning was selected to manufacture the nanofibrous barrier layer. Four different solvent compositions were tested in terms of their abilities to enable nanofibre production. The biocompatibility and the barrier properties of the electrospun layer were tested over four weeks in vitro by histological staining.

## 2. Materials

Pentaerythritol (98%), ε-caprolactone, tin (II) 2-ethylhexanoate, triethylamine (TEA), methacrylic anhydride (MAA), photoinitiator (PI) (2,4,6-trimethylbenzoyl phosphine oxide/2-hydroxy-2-methylpropiophenone blend), fungizone, fetal calf serum (FCS), penicillin/streptomycin (PS), L-glutamine, trypsin, 37% formaldehyde (FA) solution, resazurin sodium salt, glutaraldehyde, ethanol, hexamethyldisilazane (HMDS), perchloric acid, picric acid, hematoxylin solution, eosin Y solution, porcine gelatine, beta-glycerolphosphate (βGP), ascorbic acid 2-phosphate (AA2P), PCL (M_n_: 80.000 g/mol), Triton X-100, Alizarin red S, polydimethylsiloxane (PDMS, silicone), and Dulbecco’s modified Eagle’s medium (DMEM) were purchased from Sigma Aldrich (Poole, UK). Direct Red 80 (Sirius Red) was purchased from Fluka (Buchs, Switzerland). Acetone, dimethylformamide (DMF), chloroform, and industrial methylated spirit (IMS), dichloromethane (DCM), and methanol were purchased from Fisher Scientific (Pittsburgh, PA, USA). The surfactant Hypermer B246-SO-M was received as a sample from Croda (Goole, UK). Conditioning minimum essential alpha medium (α-MEM) was purchased from Lonza (Slough, UK). 4’,6-diamidino-2-phenylindole (DAPI) solution and phalloidin tetramethylrhodamine (TRITC) were purchased from ThermoFisher Scientific (San Jose, CA, USA). Optimum cutting temperature tissue freezing medium (OCT-TFM) was purchased from Leica Biosystems (Newcastle, UK). Collagenase A was purchased from Roche (Indianapolis, IN, USA).

## 3. Methods

### 3.1. Manufacturing of Polycaprolactone Polymerised High Internal Phase Emulsion (PCL PolyHIPE), PCL Electrospun, and Bilayer Membrane

#### 3.1.1. Synthesis of PCL Methacrylate

**Figure 1:**
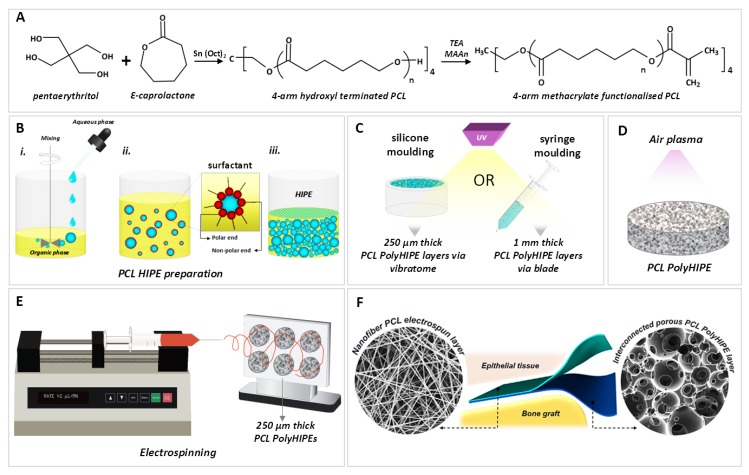
Summary of the manufacturing process of the bilayer membrane. (**A**) Synthesis of 4-arm hydroxyl-terminated polycaprolactone (PCL) and methacrylate terminalisation reaction (**B**) PCL polymerised high internal phase emulsion (polyHIPE) preparation process, (**C**) the polymerisation of PCL HIPEs to obtain PCL polyHIPE and slicing the samples, (**D**) air plasma treatment of PCL polyHIPE, (**E**) electrospinning of PCL on 250 µm thick PCL polyHIPE layer, (**F**) final representation of the bilayer barrier membrane (BM).

The PCL used in this study is 4-arm PCL methacrylate (4PCLMA), and the detailed synthesis of 4PCLMA (Figure 1A) has been described elsewhere [46]. Throughout the paper, the term “PCL polyHIPE” will be used to describe 4PCLMA polyHIPE unless otherwise stated.

Briefly, pentaerythritol and ε-caprolactone were mixed in a round flask at 160 °C while stirring continuously at 200 rpm. When pentaerythritol was dissolved, tin (II) 2-ethylhexanoate was added, and the system was removed from the oil bath to cool down. Resulting 4-arm hydroxyl terminated PCL was dissolved in DCM, and then TEA was added. The flask was placed in an ice bath. MAA was dissolved in DCM and transferred into a dropping funnel. When the addition of MAA was completed, the ice bath was removed, and the system was kept at the room temperature (RT) overnight with stirring at 375 rpm. To remove the TEA, MAA and salts formed, the methacrylated PCL was washed with HCl solution, and then with pure deionized water. Almost all solvent was evaporated using a rotary evaporator. Three methanol washes were applied, and any remaining solvent was removed by using a rotary evaporator. 4PCLMA was stored in an appropriate vessel in the freezer (−20 °C) for further use.

#### 3.1.2. Preparation of PCL HIPEs

4PCLMA (0.4 g) and the surfactant Hypermer (10% w/w of polymer) were added to a glass vial and heated to 40 °C to dissolve surfactant which is crucial for emulsion stability. Solvent blend (150% w/w of polymer, 80% chloroform, 20% toluene (w/w)) and PI (10% w/w of polymer) were added in 4PCLMA-surfactant mixture, respectively and mixed at 375 rpm using magnetic stirrer for 1 min at RT. Once the homogeneous mixture formed, 2.5 mL of water (internal phase volume 85% v/v) was added dropwise in 2 min and the emulsion was mixed further 2 min more, as illustrated in Figure 1B.

#### 3.1.3. Optimisation of Manufacturing of PCL PolyHIPEs

The emulsion templating technique was selected due to its ability to manufacture scaffolds with interconnected architecture. However, during the polymerisation, the material in contact with emulsion has been reported to have a significant effect on polyHIPE morphology [55]. 

To find the best manufacturing method in terms of creating interconnected scaffolds, we polymerised PCL HIPEs in PDMS moulds, with the upper surface in contact with air, glass, and PDMS, and we investigated the morphology of the surface and transverse sections with a scanning electron microscope (SEM).

#### 3.1.4. Manufacturing of the PCL PolyHIPE Layer

PCL HIPEs were manufactured by either polymerisation in silicone moulding and sectioning of 250 µm samples using a vibratome (Bio-Rad Polaron Division) or syringe moulding and sectioning of 1 mm samples using a scalpel. For the fabrication of a bilayer BM, 250 µm sections of PCL polyHIPE were used. Samples of 1 mm thick PCL polyHIPE were used alone for MLO-A5 cell culture, measurements of their metabolic activity, Alizarin red and Sirius red staining, histological evaluation of infiltration of murine long-bone osteocytes (MLO-A5s) and CAM experiments.

Briefly, PCL HIPE was pipetted into either silicon templates or 2.5 mL syringes (diameter of 6 mm) and cured 3 min to both sides using an ultraviolet (UV) curing system (Omnicure Series 1000, Lumen Dynamics, Canada) (Figure 1C). The resulting parts were recovered, soaked in 100% methanol for 24 h with four changes to remove any remaining contaminants of surfactant, solvent or uncured material. Then the samples were left in methanol (50% (v/v) in water) for 24 h and in water for a further 24 h. Finally, the samples were taken out from the water and left in the freezer (−80 °C) for 1 h then transferred into a vacuum oven and left for a day to preserve the porous structure of PCL polyHIPE without any collapse. 

For the fabrication of bilayer BM, 250 µm sections of PCL polyHIPE layer were obtained using a vibratome (Bio-Rad Polaron Division). For metabolic activity, Alizarin red, Sirius red, and CAM experiments, 1 mm sections of dry samples (obtained from syringe moulding) were taken using a scalpel, and these monolayer PCL polyHIPE samples were used. 

Air plasma (Diener Electronic, Ebhausen, Germany) was applied on both surfaces of the PCL polyHIPE with a power of 50 W and a pressure of 0.8 mbar for 60 s to improve cell attachment to the hydrophobic surfaces as demonstrated in our previous work (Figure 1D) [56].

#### 3.1.5. Assessment of Solvent Compositions in Terms of Their Ability to Form the Nanofibrous Structure

Four solvent compositions were investigated in terms of their ability to form the nanofibrous structure. PCL (10% (w/w)) pellets were dissolved in acetone (100%), acetone:chloroform (30:70 w/w), DCM:methanol (90:10 w/w), and chloroform:DMF (70:30 w/w). The mixtures were magnetically stirred overnight.

Solutions (~5 mL) were loaded into 5 mL syringes fitted with 0.6 mm inner diameter (ID) blunt syringe tips. The syringe was then placed in a syringe pump (Genie^TM^Plus, KentScientific, CT, USA). Aluminium foil was used as the collector and placed at a distance of 17 cm from the needle tips. The pump was set to 40 µL/min, and 17 kV voltage was applied both to the collector and the tips. Solutions of PCL prepared with various solvent blends were then electrospun at RT for 40 min.

Single layers of electrospun PCL (without polyHIPE layer) manufactured using each polymer solutions were morphologically investigated, as explained in Section 4.5. In the rest of the text, the following nomenclatures are used for electrospinning groups. Acetone (100) defines acetone (100%). Acetone:chloroform (30:70) refers to acetone:chloroform (30:70 w/w). DCM:methanol (90:10) denotes DCM:methanol (90:10 w/w), and chloroform:DMF (70:30) refers to chloroform:DMF (70:30 w/w).

#### 3.1.6. Manufacturing of Bilayer PCL Barrier Membrane (BM)

The aluminium foil collector was sprayed with methanol, and 250 µm thick sections of PCL polyHIPE layer were placed onto it. This step was performed immediately before electrospinning of the PCL barrier layer. Chloroform:DMF (70:30) solvent blend was used for the production of PCL electrospun barrier layer as explained in Section 4.5. 10% PCL solution was loaded into 5 mL syringes fitted with 0.6 mm ID blunt syringe tip. PCL was then electrospun onto PCL polyHIPE layers with a rate of 40 µL/min and a voltage of 17 kV for 40 min (Figure 1E,F).

### 3.2. Morphological, Mechanical and Surface Characterisation

#### 3.2.1. Morphological Characterisation

The micro-architectures of PCL polyHIPE, PCL electrospun, and bilayer BM were examined using an SEM. All samples were gold-coated with a voltage of 15 kV for 2.5 min using a gold sputter coater (Edwards sputter coater S150B, Crawley, UK) to increase conductivity. SEM (Philips/FEI XL-20 SEM; Cambridge, UK) was used with 10 kV power.

SEM images of the PCL fibres and PCL polyHIPE were analysed for the determination of the fibre diameters, pore size distributions, and window size using ImageJ software (Bethesda, MD, USA). Total of 54 different fibre diameters and 54 pore sizes were measured for each group of PCL electrospun layers, 100 pores and 150 windows were measured for PCL polyHIPE. All measurements were taken from three different areas of three different samples.

#### 3.2.2. Mechanical Characterisation

The bilayer BM was mechanically tested under dry and wet conditions using a mechanical testing unit (BOSE Electroforce Test Instruments, MN, USA) equipped with a 22.5 N load cell. Briefly, mechanical testing samples were cut into 10 mm × 3 mm pieces and clamped to the device with two tensile grips, and the tensile tests were performed on each sample at a rate of 0.1 mm/s until the samples failed. Elastic modulus (E), ultimate tensile strength (UTS) and elongation (%) values were calculated from stress (σ) and stress (ε) curves of each sample. The elastic modulus was determined as the slope of the initial linear section of the curve. UTS was obtained from the curve as the maximum stress that the samples could withstand. Ultimate elongation was measured as the percentage elongation of the samples at the break.

#### 3.2.3. Contact Angle Measurements

Contact angle measurements were conducted to evaluate the effect of air plasma treatment on the hydrophilicity of PCL polyHIPE. In brief, a 5 µL water droplet was dropped onto the surface of either non-treated or plasma-treated PCL polyHIPE, and the water contact angles were determined via drop shaper analyser (Krüss DSA100, Hamburg, Germany) under ambient laboratory conditions. 

### 3.3. Biological Assessment

#### 3.3.1. Cell Culture of Human Dermal Fibroblasts (HDFs)

HDFs were isolated from skin grafts taken from patients using a well-established protocol [57]. Briefly, the dermis was minced into 10 mm^2^ pieces, and the pieces were incubated overnight at 37 °C in 0.5% (w/v) collagenase A solution. The cell suspension was then centrifuged at 1000 rpm for 5 mins and resuspended and cultured in DMEM containing 10% (v/v) FBS, 100 IU mL^–1^ penicillin, 100 mg mL^–1^ streptomycin, 2 mM L-glutamine and 0.625 μg mL^–1^ amphotericin B. HDFs were used between passage 4–8. The investigations were carried out following the rules of the Declaration of Helsinki of 1975. Ethical approval for the tissue acquisition was granted by the National Research Ethics Service (NRES) Committee Yorkshire and The Humber–Sheffield (REC ref.: 15/YH/0177, REC opinion date: 03/06/2015).

#### 3.3.2. HDF Cell Seeding onto the PCL Electrospun Layer

Bilayer BMs were used as test samples to measure the metabolic activity and for histological assessment of HDFs. BMs were cut into 10 mm circles using a biopsy punch (Stiefel, Slough, UK) and 70% ethanol solution was used as an antiseptic agent for 45 min prior to cell seeding. 2 × 10^4^ HDFs were trypsinized, centrifuged, and resuspended in 100 µL of DMEM growth medium and pipetted on PCL electrospun (barrier) side of the bilayer BM. Before submerging the BMs in HDF culture medium, they were incubated at 37 °C for 2 h to allow HDFs to attach. BMs were kept in culture for 4 weeks by changing the culture medium every 2 days.

#### 3.3.3. Cell Culture of Murine Long-Bone Osteocytes (MLO-A5)

MLO-A5, murine osteoblast cell line (kindly donated by Dr Lynda Bonewald) was used to evaluate the potential of PCL polyHIPE as GBR membrane as it was previously used for evaluation of bone tissue engineering applications [47]. The T75 flasks were coated with 0.1% gelatin solution for 2 h at 37 °C and washed gently with phosphate-buffered saline (PBS) prior to cell culture. Cells were expanded on gelatine-coated T75 flasks in basal media containing α-MEM supplemented with 10% fetal bovine serum, 2 mM L-glutamine and 100 mg/mL penicillin/streptomycin. MLO-A5s cultured until 90% confluence and media was changed in every 2–3 days. Cells were used between passages 35–36.

#### 3.3.4. MLO-A5 Cell Seeding onto the PCL PolyHIPE Layer

To be able to test the full infiltration capacity of MLO-A5s through PCL polyHIPE, monolayer, 1 mm PCL polyHIPE samples (without electrospun layer) were used for biological assessment of PCL polyHIPE.

Prior to cell seeding, PCL polyHIPEs were left in 70% ethanol for 2 h and then transferred into PBS in sterile conditions, 4 washes were applied in 24 h to replace the ethanol with PBS. Finally, they were conditioned with basal media for an hour in the incubator in 24-well plates to remove the PBS completely and not to dilute the media used during the cell seeding stage with PBS. MLO-A5s were trypsinized, counted, and centrifuged. The cell pellet was re-suspended in fresh basal media (2.5 × 10^4^ cells in 20 µm). The cell suspension was placed over the surface of each PCL polyHIPE homogenously. Before polyHIPE layers were moved to the fresh wells, and 2 mL basal media was supplied into the wells, they were left for 2 h in the incubator (37.5 °C, 5% CO_2_) for cell attachment. 2 mL of media was supplied. A day after, basal media was replaced with supplemented media consisting of basal media supplemented with 5 mM βGP and 50 μg/mL AA2P. Media was changed every 2–3days.

#### 3.3.5. Assessment of Metabolic Activity

AlamarBlue® assay was performed in order to track the metabolic activities of HDFs on the PCL electrospun and MLO-A5s on PCL polyHIPE. 0.1 mM AlamarBlue® working solution was prepared by 10× dilution of the 1 mM AlamarBlue® stock solution with growth medium. At days 1, 7, 14, 21, and 28 growth media were removed, and the samples were washed with PBS. 1 mL of AlamarBlue® working solution was added to each well and incubated at 37 °C for 4 h. After an incubation period, 200 µL of the solution was transferred into a 96-well plate, and the fluorescence readings were done at an excitation wavelength of 540 nm and an emission wavelength of 635 nm. Fresh samples were used for the measurements at each time point.

#### 3.3.6. Assessment of Calcium Deposition

Alizarin red staining was performed to assess the calcium deposition of MLO-A5s on PCL polyHIPE. Briefly, Alizarin red powder was dissolved in deionized water at 1 w/v% in a water bath and filtered to remove particles to make Alizarin red solution (ARS). PCL polyHIPEs were submerged in 1 mL of ARS solution and incubated for 1 h. ARS solution was removed, and the samples were washed every 5 min with deionized water and gentle orbital shaking until the water remains clear. They were submerged with 1 mL of 5% perchloric acid to destain and left for further 30 min with gentle orbital shaking; 150 μL of the destain solution in triplicates were transferred into a clear 96-well plate and read at an absorbance of 405 nm.

#### 3.3.7. Assessment of Collagen Deposition

Sirius red staining was performed to assess the collagen deposition of MLO-A5s on PCL polyHIPE. Briefly, Sirius red (direct 80) powder was dissolved in saturated picric acid (1 w/v%) to form Sirius red solution (SRS) and filtered to ensure no particles remain. PCL polyHIPEs were submerged with 1 mL of SRS solution and left for 1 h. SRS solution was removed, and the samples were washed every five min with deionized water and gentle orbital shaking until the water remains clear. They were submerged with 1 mL of 0.2 M sodium hydroxide (NaOH):methanol (1:1) to destain and left for 30 min with gentle orbital shaking. 150 μL of the destain solution in triplicates were transferred into a clear 96-well plate and read at an absorbance of 405 nm.

#### 3.3.8. Haematoxylin and Eosin (H&E) and Alizarin Red Staining

Bilayer BM and PCL polyHIPE cultured with HDFs and MLO-A5s, respectively for 1-week and 4-week, and PCL polyHIPE on CAM were stained with haematoxylin and eosin (H&E) using a standard protocol [58]. Briefly, samples were washed with PBS before (once) and after (three times) fixing them in 3.7% FA for 30 min at RT. Meanwhile, cryomoulds were filled with OCT-TFM. Samples were embedded in it, and the rest of the volume was then filled with OCT-TFM to the top. Cryomoulds were placed into liquid nitrogen and incubated for 5–7 min until solidified. Frozen blocks were fixed on mounting platforms, and placed into a cryostat (Leica CM1860 UV, Milton Keynes, UK) before sections were sliced at 5–10 µm and immediately mounted onto the surface of Thermo SuperFrost® Plus slides. For H&E staining, slides were stained with hematoxylin for 90 s and eosin for 5 min. For calcium staining, slides were stained with 2% (w/v, in water) ARS for 5 min. Excess dye was shaken off, and the slides were rinsed, dehydrated, cleared and mounted the slide using the permanent mounting medium.

#### 3.3.9. Preparation of Biological Samples for Scanning Electron Microscope (SEM)

On day 28, the PCL polyHIPE discs seeded with MLO-A5s were washed 3 times with PBS and fixed with 2.5% glutaraldehyde at RT for 1 h and rinsed with PBS. Then the discs were soaked in deionised water for 5 min prior to dehydration of the samples with serial ethanol washes. Finally, HMDS is used as the chemical drying agent, and the discs were soaked in HMDS:ethanol (1:1) solution for 1 h and transferred into 100% HMDS for 5 min. The samples were then air-dried overnight in a fume hood and gold-coated at a current of 15 mA for 2.5 min with a gold sputter (Edwards sputter coater S150B, Crawley, England) prior to imaging under SEM (Philips/FEI XL-20 SEM; Cambridge, UK).

#### 3.3.10. Fluorescent Staining

At days 7 and 28, PCL polyHIPE discs were fixed with 3.7% FA for 30 min and washed gently with PBS prior to submerging into 0.1% (v/v) Triton X 100 (in PBS) solution for 20 min. After serial PBS washes, phalloidin-TRITC (1:500 diluted in PBS from stock solution) solution was added onto samples to visualize F-actin filaments of the cells and incubated for 30 min at RT in the dark. Discs were washed 3 times with PBS. To stain the cell nuclei, DAPI solution (1:1000 diluted in PBS) was added onto the polyHIPE discs and incubated for 10–15 min at RT in the dark; samples were then washed 3 times with PBS and imaged under a fluorescent microscope (Olympus IX3, Tokyo, Japan).

#### 3.3.11. Ex-ovo Chorioallantoic Membrane (CAM) Assay

An ex-ovo CAM assay was used to evaluate the potential of PCL polyHIPE layer for the suitability of blood vessel ingrowth, as described previously [59,60]. Briefly, fertilised chicken eggs (Gallus Domesticus) were purchased from Henry Stewart & Co. MedEggs (Norwich, UK) and cleaned with 20% IMS solution. Eggs were incubated at 37.5 °C for 3 days in an egg incubator (RCOM King SURO, P&T Poultry, Powys, Wales, UK). At the end of day 3, the embryos were transferred gently into sterile Petri dishes and incubated at 38 °C in a humidified cell culture incubator (Binder, Tuttlingen, Germany). On day 7, PCL polyHIPE discs were implanted to CAM, and the chicks were incubated for further 7 days. On day 14, the chicks were euthanized, and the CAMs with PCL polyHIPE integrated to them were removed and fixed in 3.7% FA solution. Sections of the CAMs were taken and stained with H&E as described in Section 3.3.8.

### 3.4. Statistical Analysis

Statistical analysis was carried out using one-way and two-way analysis of variance (ANOVA) using statistical analysis software (GraphPad Prism, CA, USA). Where relevant, n values are given in figure captions. Error bars indicate standard deviations in the graphs unless otherwise stated.

## 4. Results and Discussion

### 4.1. Manufacturing and Characterization of the PCL PolyHIPE Layer

**Figure 2:**
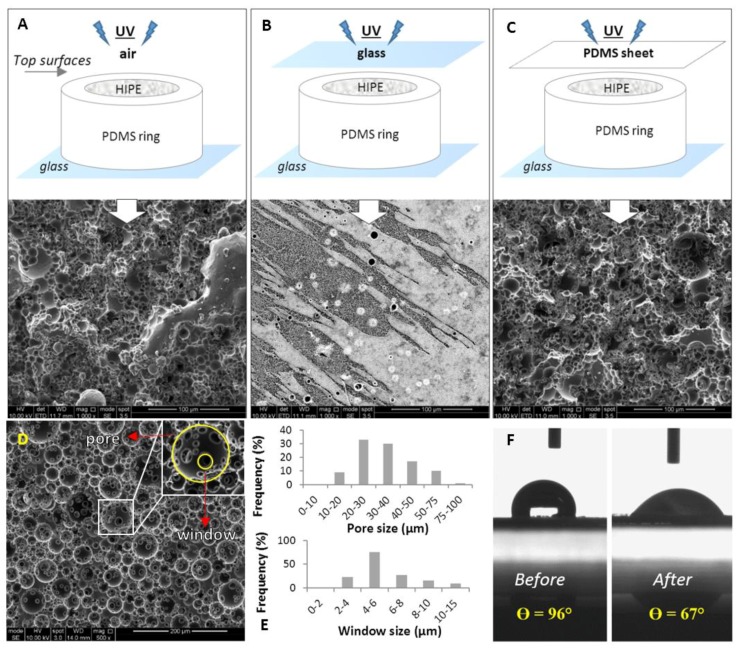
Scanning electron microscope (SEM) images of the top surfaces PCL polyHIPEs cured in contact with; (**A**) air, (**B**) glass, and (**C**) polydimethylsiloxane (PDMS) sheet. (**D**) SEM image of the transverse section of PCL polyHIPEs. (**E**) Pore size and window size distributions of the inner section. (**F**) Contact angle measurements of a water droplet on PCL polyHIPE before and after air plasma treatment (n = 3).

The surface of PCL polyHIPEs polymerized in contact with air, glass, or PDMS showed different morphologies (Figure 2A–C). When the surface was not covered by any substrate, and UV was directly applied on PCL HIPEs, the surface was porous, but it did not have open interconnected cellular morphology (Figure 2A). When the surface of the HIPE was in contact with glass, the surface showed microscale roughness, rather than pores (Figure 2B). In terms of interconnectivity, the best surface morphology was obtained when the PDMS sheet was used as a cover. PCL polyHIPE surfaces created this way had a mixture of open and closed porous morphology (Figure 2C). 

The significant influence of the mould material on polyHIPE has been reported previously [55]. This study correlated the surface interconnectivity with the following potential scenarios on the polyHIPE–mould interface; (i) polyHIPE can potentially bind to mould surface leading to difficulties in demoulding, (ii) the mould can leach materials leading to contamination of the polyHIPE surface, and (iii) partial phase separation of the emulsion which leads to closed-pore polyHIPE surfaces.

Figure 2D shows the transverse section of PCL polyHIPE. It has a homogenous, open cellular architecture with interconnected porosity. Pore interconnects are pathways for cells, waste and nutrients, the interconnectivity of the scaffold is a crucial feature for cell invasion, tissue integration and vascularisation [61,62,63,64]. To be able to benefit from the interconnected inner morphology of the scaffolds, it was decided to create the PCL polyHIPE layer by sectioning bulk pieces into slices as described in Section 3.1.4.

The pore sizes of the PCL polyHIPE layer were distributed between 10–78 µm; the average pore size (D) was found 34 ± 13 µm, 90% of the pores have the pore sizes between 20–75 µm range (Figure 2E). The window sizes were distributed between the 2–13 µm range, and the average window size (d) was measured as 6 ± 2 µm (Figure 2E), which gives the degree of connectivity (d/D) as 0.18. In our previous study, when the same solvent composition was used to dilute PCL (80:20 chloroform:toluene (w/w)) the average pore size and the window size was found to be 20 ± 7 µm and 4 ± 2, respectively [46]. The difference between the pore and window size found in the previous study and the current work can be explained with the two main compositional changes; (i) increasing the internal phase volume from 82% to 85%, and (ii) increasing the total solvent volume from 0.40 mL to 0.46 mL. A higher internal phase volume is expected to reduce the average pore size while increasing the average window diameter as water droplets will need to be more tightly packed. On the other side, the increasing solvent amount is expected to show a dramatic increase in the average pore diameter [65]. The overall effect of these two compositional changes resulted in approximately 50% increase in average pore size and window diameter.

The main drawback of using PCL for tissue engineering scaffold material is its hydrophobicity, which limits cell attachment to the material surface. To overcome this, oxidising the surface by plasma treatment is one of the most popular methods for enhancing cell attachment [56,66,67,68,69,70]. In this study, our finding also proved that air plasma treatment changes the surfaces from hydrophobic to hydrophilic and this change encourages the cell attachment and cellular infiltration on the PCL polyHIPE layer which will be further discussed following sections. Contact angles of the water droplets on non-treated (P-) and air plasma treated (P+) PCL polyHIPEs were measured as 96° ± 4° and 67° ± 4°, respectively (Figure 2F).

### 4.2. Assessment of the Metabolic Activity of MLO-A5s on PCL PolyHIPE and the Cellular Infiltration through PCL PolyHIPE Layer

**Figure 3:**
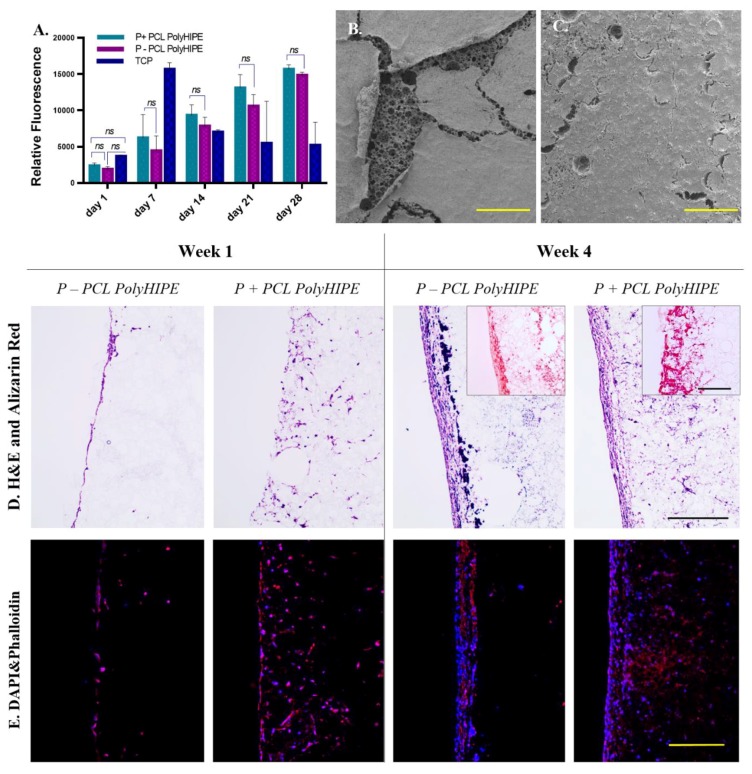
(**A**) Metabolic activity of murine long-bone osteocytes (MLO-A5s) cultured on P-, P+ PCL polyHIPEs, and tissue culture plate (TCP) for 4 weeks. SEM images of the top surfaces of (**B**) P+ and (**C**) P- PCL polyHIPEs cultured MLO-A5s on for 4 weeks (Scale bar represents 500 µm). (**D**) Haematoxylin and eosin (H&E) and Alizarin red, and (**E**) fluorescent staining of MLO-A5s cultured on P+ and P- PCL polyHIPEs for 1 week and 4 weeks (Scale bar represents 250 µm, blue: 4’,6-diamidino-2-phenylindole (DAPI), red: phalloidin tetramethylrhodamine (TRITC)).

At all-time points, the metabolic activity of MLO-A5s cultured on P+ PCL polyHIPEs was slightly higher than MLO-A5s cultured on P- PCL polyHIPEs, but there was no statistical difference observed between these two groups (Figure 3A). Metabolic activities of MLO-A5s on both P+ and P- PCL polyHIPEs increase from day 1 to day 28 gradually, but the dramatic decrease was observed in the metabolic activity of the MLO-A5s on tissue culture plate (TCP) after day 7 which is also discussed in Section 4.7.

Figure 3B,C clearly show the positive impact of air plasma treatment of PCL polyHIPE on the attachment of MLO-A5s to the surface at day 28. While the layer of MLO-A5s is peeled off from the surface of P- PCL polyHIPE, cells on P+ PCL polyHIPE are still integrated with the polyHIPE layer. The preparation steps of the biological samples for SEM includes multiple washing steps and drying (Section 3.3.9). The loosely attached cell layer detached from P- PCL polyHIPE at the end of all these steps, probably due to limited cell penetration into the pores.

Although air plasma treatment seems as it has not had a significant effect on the metabolic activity of MLO-A5s, H&E and fluorescent images support the finding from SEM images, and they show that air plasma treatment has a huge impact on cell infiltration (Figure 3D,E). At week 1, while MLO-A5s only accumulated on the surface of the P- PCL polyHIPE with nearly no infiltration, they were observed as migrating through the pores the P+ PCL polyHIPE. 

Even during the seeding of the MLO-A5s on the PCL polyHIPE layer, the positive effect of plasma treatment was observed. Once the cell suspension was placed on the top of the PCL polyHIPE, it was immediately absorbed by P+ polyHIPE but stayed as a droplet on the P- layer. This indicates that even from the cell-seeding stage onwards, plasma treatment encourages cells to migrate into the pores of the PCL polyHIPE layer. Although MLO-A5s tend to densely accumulate on the top of both PCL polyHIPEs at week 4, cell migration up to 400 µm was observed on P+ polyHIPEs. This positive influence of air plasma treatment on polymer scaffold has also been demonstrated in vivo. Valence et al. had reported improvement of cell attachment and infiltration within a vascular graft upon plasma treatment when materials were implanted subcutaneously [25].

Interestingly, on H&E slides, very small-sized haematoxylin-stained particles (different than haematoxylin stained cells) were observed only at week 4 at both P+ and P- PCL polyHIPEs (Figure 3D). Fluorescent staining shows that they are not cells. It has been previously reported that haematoxylin selectivity stains calcium-containing particles [71]. Alizarin red staining images shows densely accumulated calcium on the top of P- PCL polyHIPE and comparably less dense stains in deeper pores, while there is dense calcium deposition P+ PCL polyHIPE up to 400 µm deep (Figure 3D).

### 4.3. Assessment of the Extracellular Matrix (ECM) Deposition of MLO-A5s on PCL PolyHIPE Layer

As MLO-A5s cultured in supplemented media, they were expected to deposit calcified extracellular matrix (ECM) [47,72,73]. Prideaux et al. previously reported that supplementation of MLO-A5 cell cultures with AA2P and βGP showed a significant increase in ECM mineralization compared to the non-supplemented group [14].

**Figure 4:**
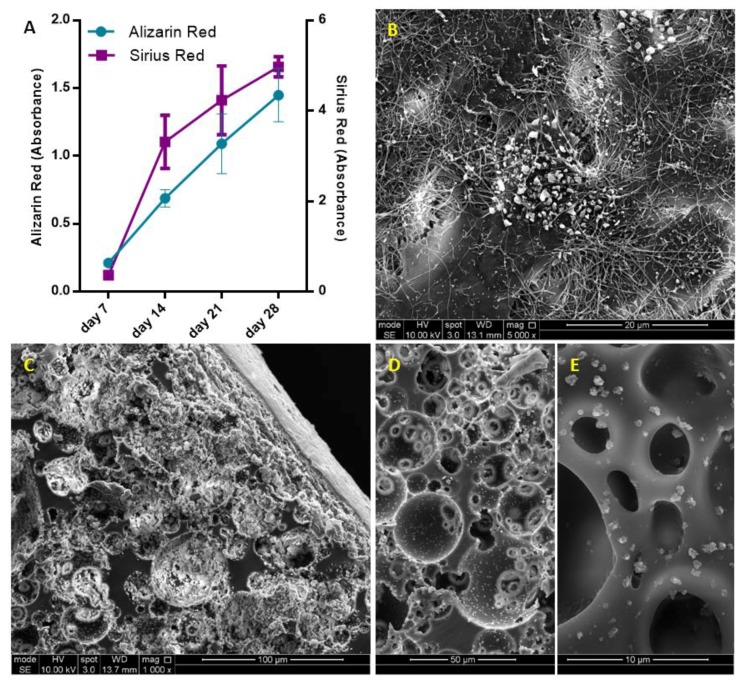
(**A**) Assessment of calcium and collagen deposition of MLO-A5s after 7, 14, 21 and 28-day culture on PCL polyHIPE by using Alizarin red and Sirius red, respectively. (**B**) Surface (**C**–**E**) cross-section of PCL polyHIPE cultured with MLO-A5s for 28 days in supplemented media.

Calcium and collagen deposition on P+ PCL polyHIPE gradually increased from day 7 to day 28 (Figure 4A) (All subsequent studies were conducted on P+ PCL polyHIPE only). ECM deposition, mineral nodules, and collagen fibres of MLO-A5s cultured on PCL polyHIPE layer for 4 weeks are shown in Figure 4B. An SEM image of the cross-section of the PCL polyHIPE shows the pores densely filled with cells and extracellular material (Figure 4C). Additionally, sub-micrometric crystalline debris was observed in regions beyond the maximum cell ingrowth (Figure 4D,E), these indicate the existence of calcium deposits deep within the polyHIPE layer, as also observed in on H&E and Alizarin red images.

These calcium deposits look similar to surfaces of polyHIPE layer incubated in simulated body fluid, which is commonly used to test the ability of the formation of bone-like apatite or mineral deposition on scaffolds [74,75,76]. The source and mechanism of the formation of the deposited calcium-containing crystals will be investigated in future studies.

### 4.4. Assessment of the Performance of PCL PolyHIPE for Supporting Blood Vessel Ingrowth Using Ex-ovo CAM Assay

**Figure 5:**
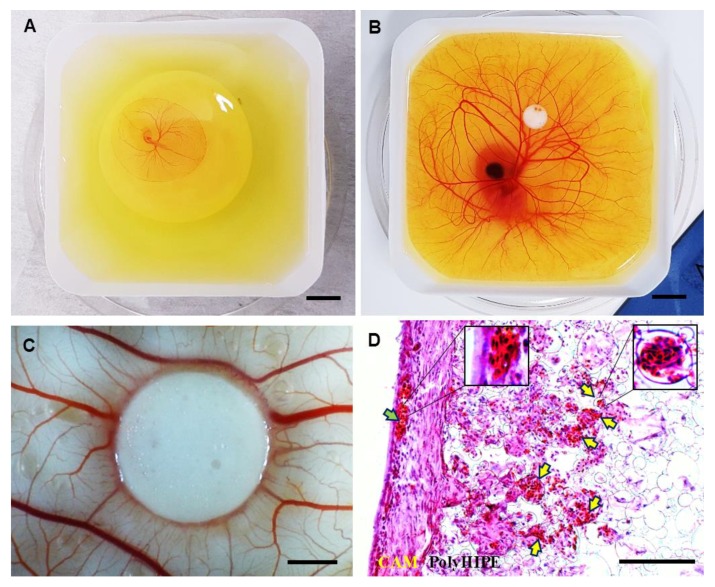
Chick embryos in a petri dish on (**A**) embryonic development day 3 and (**B**) embryonic development day 7 (Scale bar represents 10 mm). (**C**) PCL polyHIPE on chorioallantoic membrane (CAM) at day 14 (Scale bar represents 2 mm). (**D**) H&E image of PCL polyHIPE on CAM at day 14. (Green arrow indicates the blood vessel on the CAM itself; yellow arrows indicate the blood vessels in PCL polyHIPE (Scale bar represents 100 µm).

The CAM assay is a well-established method for the assessment of angiogenesis and initial response to biomaterials [59,60,77]. In an ex-ovo CAM assay, the embryos are transferred into petri dish on day 3 (Figure 5A) and incubated until day 7 (Figure 5B) which is the day of material implantation. At day 14, the following features can be assessed macroscopically (Figure 5C) and histologically (Figure 5D): (i) biocompatibility, (ii) cellular infiltration capacity and (iii) the performance of the PCL polyHIPE layer for supporting vascularisation. 

Our laboratory has reported the average survival rate for the ex-ovo CAM assay as 68% for intermediate and 83% for experienced users [60]. In this study, the survival rate of the chicks was approximately 75% and 73% for non-implanted and PCL polyHIPE implanted groups, respectively, in line with previous investigations. Thus, the PCL polyHIPE showed good biocompatibility, and the implantation of the material did not affect the survival rate of the chicks. 

The integration of the CAM tissue into PCL polyHIPE was examined. Extensive cell infiltration was observed from the CAM tissue to PCL polyHIPE, showing complete integration of the material with the CAM. During the isolation of the PCL polyHIPE from the CAM, it was not possible to separate it from the CAM, which is also an indication of strong integration. This is in line with studies reported by other groups on the good-integration of PCL porous scaffolds with CAM [78,79,80]. The infiltration capacity of the cells into PCL polyHIPE was better in the ex-ovo CAM assay (Figure 5D) when compared with the in vitro histology data (Figure 3D). This is potentially due to the continuous contact of the PCL polyHIPE with a dense and dynamic cell population in the CAM.

Assessment of the polyHIPE material on the CAM demonstrated that the structure and the pore size of the polyHIPE were suitable for supporting blood vessel ingrowth through the polyHIPE. H&E staining shows that alongside the high level of integration of the host CAM tissue with the polyHIPE layer, many blood vessels were found growing into the pores of PCL polyHIPE and through the interconnects (Figure 5D) in only 7 days. 

Current understanding of vascularisation of porous scaffolds indicates that the pore size should be at least 250 µm for vascularisation to occur [81,82], but some studies suggest smaller pore sizes can also allow for the ingrowth of blood vessels. Madden et al. have shown that 30–40 µm pore size with 15 µm interconnects are suitable for vascularisation in rats [83]. Similarly, Baker et al. reported that particulate-leached PCL scaffolds with 5–200 µm pore range allowed extensive vascularisation in the scaffold when implanted subcutaneously into rats [84]. Klenke et al. observed vascularization in ceramic particles with macropores ranged from 40 to 280 μm [85]. Finally, our group has demonstrated the vascularisation of polylactic acid electrospun scaffolds with a mean pore size of 4.25 µm in the CAM assay [86].

By using the CAM assay, we have shown the performance of the developed BM for supporting tissue integration and vascularisation. Both are critical factors in avoiding delay in osteogenesis and tissue regeneration and overcoming the rejection of an implant [87,88].

### 4.5. Assessment of Solvent Compositions in Terms of Their Ability to Form the Nanofibrous Structure

**Figure 6:**
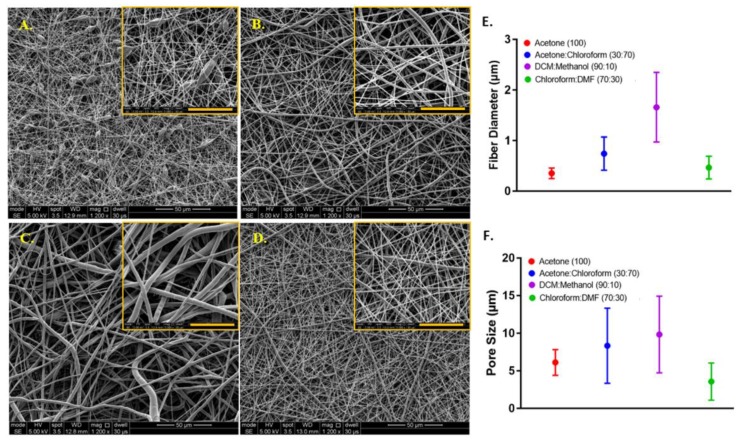
Morphological characterisation of the electrospun PCL fibres, where polymer solutions were prepared with different solvents. SEM image of PCL electrospun prepared by dissolving PCL in (**A**) acetone (100), (**B**) acetone:chloroform (30:70), (**C**) dichloromethane (DCM):methanol (90:10), (**D**) chloroform:dimethylformamide (DMF) (70:30). The graphs show (**E**) the fibre diameter and (**F**) the pore size distributions, respectively. Yellow scale bars represent 20µm.

The mean diameters of the PCL fibres where polymer solutions were prepared with different solvents were 0.35 ± 0.10 µm, 0.74 ± 0.32 µm, 1.69 ± 0.75 µm, and 0.47 ± 0.22 µm, and the average pore sizes were 6.28 ± 2.30 µm, 8.34 ± 4.96 µm, 9.84 ± 5.25 µm, and 3.57 ± 2.08 µm for acetone (100), acetone:chloroform (30:70), DCM:methanol (90:10), and chloroform:DMF (70:30) groups, respectively (Figure 6).

Except for the acetone (100) group, a decrease in the pore sizes was observed when the diameter of the PCL fibres gets smaller. Although the acetone (100) led to the formation of the smallest diameter PCL fibres, the smallest pore size was calculated for the electrospun layer prepared with chloroform:DMF (70:30).

When acetone was used as the sole solvent, it was difficult to electrospin the solution, and bead formation occurred. The undesirable bead formation during electrospinning is likely to increase pore size between the fibres [89]. One of the main reason for the formation of thinner fibres and beads has been reported as the lower viscosity of the electrospinning solution [90]. It has previously been shown that among the five solvents used in this study, acetone has the lowest viscosity [91]. Zverev et al. reported that the viscosity of the polymer solution changes with the solubility, and low viscosity is linked with poor solubility when other parameters kept constant [92].

The electrospinnability of the PCL solutions from high to low was: chloroform:DMF (70:30) > acetone:chloroform (30:70) > acetone (100) > DCM:methanol (90:10). The quality of the PCL electrospinning was assessed based on smooth fibre formation, bead or particle formation and continuous electrospinning of the solution, which depend on parameters such as solubility, viscosity, dielectric constant, and conductivity [93].

The solubility of the polymer in a solvent has a major effect on electrospinning nanofibres. DCM, methanol, chloroform, DMF and acetone (as single solvents or solvent blends) are common solvents for dissolving PCL and widely used for the production of PCL fibres with electrospinning [38,94]. Among these solvents, PCL has a higher solubility in chloroform and DCM, whereas the solubility of PCL is poor in DMF, acetone, and methanol [95]. 

When acetone was used as the single solvent to dissolve PCL, the solution resulted in poor electrospinnability and the formation of undesired beads during the electrospinning process. Using the acetone:chloroform (30:70) solvent blend significantly increased the electrospinnability of PCL, which can be explained by the addition of chloroform to the solvent mixture, in which PCL has higher solubility [96]. The ability to electrospin PCL dissolved in DCM:methanol (90:10) was very poor, and we did not manage to obtain nanofibres when this solvent used for electrospinning. This can be explained by the low dielectric constant and conductivity of the main solvent, DCM, in the solvent blend [97]. The best solvent blend for electrospinning PCL nanofibres was chloroform:DMF (70:30) solvent composition used. Although DMF is not classified as a good solvent for PCL, it has a high dielectric constant and, it is a polyelectrolyte [98]. Due et al. previously reported that the addition of DMF to the solvent blend improves the electrospinnability of PCL and leads to smaller diameter fibre formation [99]. Kanani et al. had shown that when DMF was added to methylene chloride, and the solvent mixture used for electrospinning PCL, the spinning process was improved, and uniform nanofibres were obtained [40]. Hsu et al. demonstrated a reduction in the diameter of electrospun PCL fibres with the addition of DMF to chloroform [100]. Bolgen et al. observed a dramatic decrease in diameter (from 1300 nm to 300 nm) when DMF was included in the solvent mixture up to 40% [101].

In this study, the chloroform:DMF (70:30) solvent blend was selected for the manufacturing of nanofibrous barrier layer due to multiple factors including the improved electrospinnability, the decreased fibre diameter, and the smaller pore size.

### 4.6. Manufacturing and Characterisation of the PCL Bilayer Barrier Membrane

**Figure 7:**
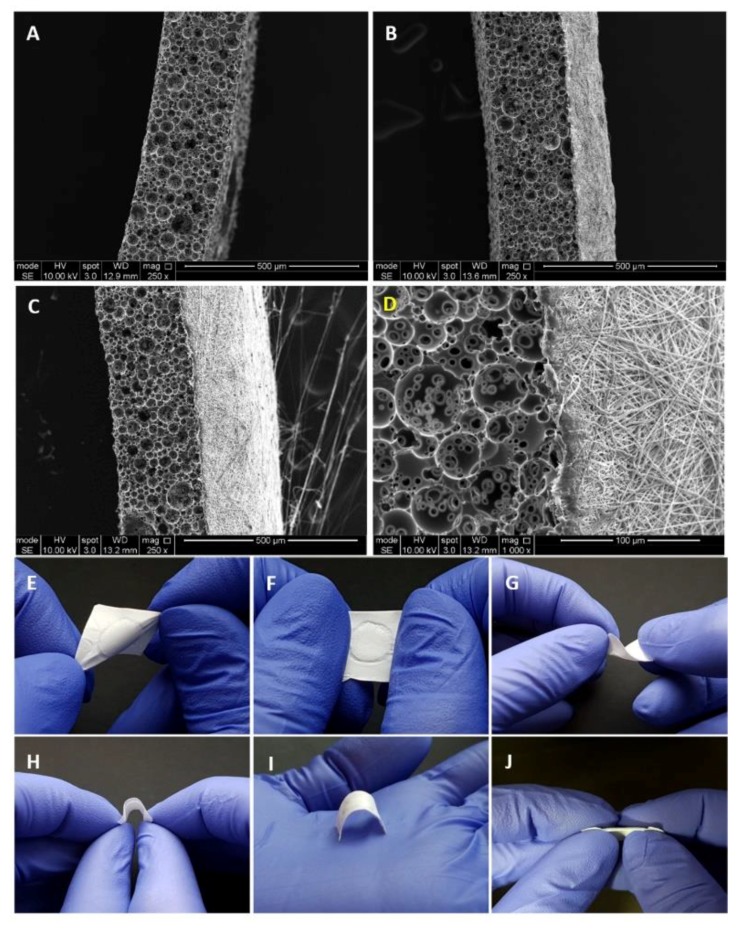
SEM images of (**A**) 250 µm sectioned PCL polyHIPE layer, (**B**) 20 min PCL electrospun on PCL polyHIPE, (**C**) 40 min PCL electrospun on PCL polyHIPE, (**D**) Higher magnification SEM image showing the border of two layers. Macro images of the bilayer PCL barrier membrane (BM) to show the suitability of the design for (**E**–**F**) stretching in different axes, (**G**–**H**) bending, (**I**) space making, and (**J**) side view of the BM to show the integration of the two layers.

Following the optimisation of manufacturing of PCL electrospun and PCL polyHIPE layers, two layers were combined to fabricate the bilayer BM (Figure 7A–D). The complete integration of both layers can be seen from SEM images. This is more likely due to the fact that both polymers are PCL, and the solvent composition used for electrospinning PCL can partially dissolve the surface of the PCL polyHIPE layer. No delamination of the two layers was observed, and the BM preserved its integrity during the experiments. 

Figure 7E–J shows the handling ability of the PCL bilayer BM. The resulting BM was very flexible and allowed manual handling, including bending and twisting without losing its structural integrity. Figure 7I shows the space making ability of the BM, which is defined as the ability to maintain a space for cells without any collapse.

For this study, the thicknesses of the PCL electrospun and PCL polyHIPE layers were determined as 200 µm and 250 µm, respectively. The thicknesses of the PCL electrospun and PCL polyHIPE layers can be controlled easily by changing the electrospinning time and slicing thickness, respectively. To show the controllability of the thickness of the PCL electrospun layer, Figure 7B shows a bilayer membrane with a low thickness where PCL was electrospun on polyHIPE for 20 min instead of 40 min. Thicker membranes are assumed to have better barrier performances in addition to higher mechanical strength [102] and a longer degradation time and which results in the GTR membrane being present during a longer time period [103]. The question of the optimum barrier membrane thickness can be answered to some extent, experimentally in vitro, but ideally, it needs to be investigated in vivo in future studies. Here, the tunability of the thickness of individual layers is an advantage in our manufacturing method as we can provide BMs of varying thicknesses for comparative evaluation of performance and rate of breakdown in vivo. 

Tensile tests of the BMs were conducted on both dry and wet conditions. Biomedical implants are usually in contact with body fluids, which significantly influences their performance in comparison with their dry state. The mechanical behaviour of an implant under wet conditions is important for better representing the in vivo conditions [104]. 

**Figure 8:**
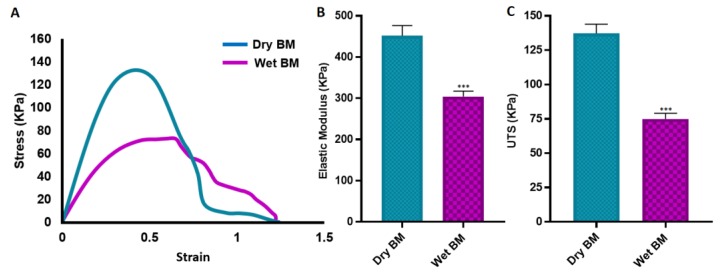
Mechanical properties of the BM under dry and wet conditions. (**A**) Representative stress-strain curves, (**B**) elastic modulus, (**C**) ultimate tensile strength (UTS) of the BMs under dry and wet conditions (*** p ≤ 0.001, n = 3).

Both the E and UTS values of dry BMs were significantly higher compared with the wet BM samples. But there was not any statistically significant difference between the elongation of the BMs in dry and wet state (Figure 8). 

The UTS of the BMs were measured as 137.3 ± 6.7 KPa and 75.0±4.2 KPa for dry and wet samples, respectively. The elastic modulus and elongation of dry and wet BMs are 452.1 ± 24.5 KPa and 304.2 ± 12.9 KPa; and 79.3 ± 3.5% and 83.2 ± 2.1%, respectively. 

The mechanical properties of the developed membrane show similarities with other developed membranes in literature. Lee et al. reported tensile strength of commercial collagen membrane (Ossix plus) of around 110 KPa and 20 KPa for the dry and wet state, respectively [102]. Poly(lactic-co-glycolic acid) (PLGA) membrane fabricated with freezing and lyophilisation has been reported to have similar UTS with our BM where the elongation of the PLGA membrane was approximately eight times lower [105]. Similarly, the tensile strength of the freeze-gelated chitosan membrane has been demonstrated approximately four times and 10 times lower, respectively under dry and wet conditions when compared with the developed BM in this study [33]. Electrospun chitosan membrane with random fibre orientation has been shown to have slightly higher UTS in a wet state, but at the same time, it was approximately 10 times less elastic than our BM, and the elongation was almost six times lower [106]. Another study has revealed that the polysaccharide/bioactive glass membrane produced using the layer by layer deposition technique has very similar mechanical properties in terms of UTS and E values with our BM [107].

### 4.7. Assessment of the Metabolic Activity of HDFs on PCL Electrospun Layer and the Ability of the PCL Electrospun Layer to Act as a Cell Barrier

**Figure 9:**
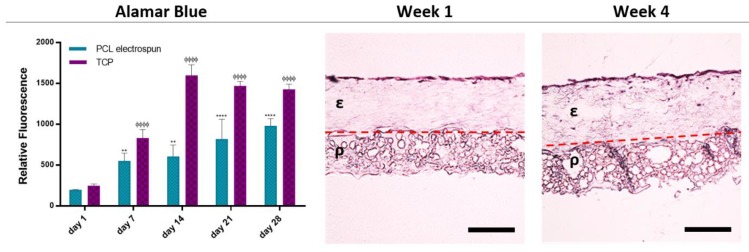
Evaluation of the biocompatibility and the barrier properties of the bilayer BM. The metabolic activity of the HDFs growing on PCL electrospun layer from day 1 to day 28 is given in the graph (*** and ΦΦΦ p ≤ 0.001, ** and ΦΦ p ≤ 0.01, * and Φ p ≤ 0.05, n = 3). Histological images demonstrate the barrier properties of the PCL electrospun layer over 4 weeks. ε and ρ indicate the electrospun layer and PCL polyHIPE layer, respectively. Dotted line indicates the boundary of the two layers (Scale bar represents 200 µm).

The metabolic activities of HDFs growing on the PCL electrospun layer gradually increased from day 1 to 28 (Figure 9) showing the biocompatibility of the bilayer PCL membrane. Although the metabolic activities of the HDFs growing on TCP were higher at each time point, they started to drop after day 14. This decrease is more likely to be due to the limited to two dimensional surface of the TCP, which restricts the capacity of cells to expand [108].

Histological analysis of the PCL electrospun layer showed that HDFs were not able to penetrate due to the small pore sizes of nanofibrous random PCL fibres. Instead, they were observed as growing on the surface of the electrospun barrier layer and not migrating towards the polyHIPE layer (Figure 9) confirming the ability of cell-occlusiveness of the electrospun PCL layer. Randomly orientated nanofibrous scaffolds have been demonstrated as a physical barrier to cell penetration while allowing the diffusion of nutrients. Previous work from our laboratory has shown that keratinocytes and fibroblasts were successfully segregated when separated by a nanofibrous poly(3-hydroxybutyrate-co-3-hydroxyvalerate) (PHBV) layer [30]. Similarly, Vaquette et al. showed that fibroblasts seeded on a random fibre mat did not penetrate the scaffold and colonized on the surface and formed a 30 µm thick cell sheet [109]. 

As the crucial time for epithelial invasion has been reported as the first 14 days of implantation, then the barrier function limiting the epithelial invasion up to 14 days is considered sufficient for GBR applications [110,111].

## 5. Conclusions

In the present study, a bilayer BM made of a biodegradable synthetic polymer, PCL, was successfully fabricated by combining electrospinning and with emulsion templating. The resulting BM showed no delamination, and its structure was qualitatively resilient to torsion and stretching, and it was straightforward to handle. The electrospun layer of the BM has been confirmed for its barrier features for the prevention of soft tissue invasion whereas the interconnected PCL polyHIPE layer has shown potential for use as the bone promoting layer providing the key requirements such as cell compatibility, supporting cellular infiltration, and promoting collagen and mineral deposition. Furthermore, the pore structure of the PCL polyHIPE layer has been found to be suitable for blood vessel ingrowth. In conclusion, by combining two methods of fabricating an FDA approved polymer, PCL, a bilayer BM that is a good candidate for a diverse range of GTR applications can be fabricated. Further exploration of the in vivo performance of the developed BM will be interesting in future studies.
